# Disentangling the Effects of Risk Factors and Clinical Care on Subnational Variation in Early Neonatal Mortality in the United States

**DOI:** 10.1371/journal.pone.0049399

**Published:** 2012-11-14

**Authors:** Lahn D. Straney, Stephen S. Lim, Christopher J. L. Murray

**Affiliations:** 1 The Institute for Health Metrics and Evaluation, The Department of Global Health, University of Washington, Seattle, Washington, United States of America; 2 Department of Epidemiology and Preventative Medicine, Monash University, Melbourne, Victoria, Australia; University of Hong Kong, China

## Abstract

**Objective:**

Between 1990 and 2010, the U.S ranking in neonatal mortality slipped from 29^th^ to 45^th^ among countries globally. Substantial subnational variation in newborn mortality also exists. Our objective is to measure the extent to which trends and subnational variation in early neonatal mortality reflect differences in the prevalence of risk factors (gestational age and birth weight) compared to differences in clinical care.

**Methods:**

Observational study using linked birth and death data for all births in the United States between 1996 and 2006. We examined health service area (HSA) level variation in the expected early neonatal mortality rate, based on gestational age (GA) and birth-weight (BW), and GA-BW adjusted mortality as a proxy for clinical care. We analyzed the relationship between selected health system indicators and GA-BW-adjusted mortality.

**Results:**

The early neonatal death (ENND) rate declined 12% between 1996 and 2006 (2.39 to 2.10 per 1000 live births). This occurred despite increases in risk factor prevalence. There was significant HSA-level variation in the expected ENND rate (Rate Ratio: 0.73–1.47) and the GA-BW adjusted rate (Rate ratio: 0.63–1.68). Accounting for preterm volume (defined as <34 weeks), the number of neonatologist and NICU beds, 25.2% and 58.7% of the HSA-level variance in outcomes was explained among all births and very low birth weight babies, respectively.

**Conclusion:**

Improvements in mortality could be realized through the expansion or reallocation of clinical neonatal resources, particularly in HSAs with a high volume of preterm births; however, prevention of preterm births and low-birth weight babies has a greater potential to improve newborn survival in the United States.

## Introduction

In 1990, the US ranked 29th among all nations with a neonatal mortality rate of 6.2/1,000 births [1]. Although the rate has declined in the US, it has been slower to decline than other countries. In 2010 the US slipped to 45^th^, with a neonatal mortality rate of 4.0/1,000, among all nations; this is in comparison to Japan which had the lowest rate globally of 1.2/1,000 [1]. As with other health indicators in the US [2], these national numbers mask significant disparities. The neonatal mortality rate in 2006 was 2.4 times higher in the District of Columbia than in Washington State [3].

Neonatal mortality can be reduced through reducing exposure to risk factors such as prenatal smoking and alcohol consumption [4]–[7], and improving access to and quality of prenatal [8], [9] obstetric and delivery, and neonatal care. Risk-adjustment models can be used to understand the contribution of these different approaches to reduce neonatal mortality [10]. For example, by controlling for risk factors such as gestational age and birth weight on admission to neonatal intensive care units (NICUs), a study in Massachusetts suggests that improvements in newborn survival have been achieved primarily through improvements in the quality of NICUs rather than healthier neonates [11]. Risk-adjusted outcomes also provide an avenue to understand variation in health system performance, and to identify what distinguishes high performers from low performers. Hospitals with a higher volume of births [12]–[17] and higher levels of care [16], [18]–[23] have been associated with lower risk-adjusted neonatal mortality.

Although several studies have examined risk-adjusted neonatal mortality, several important gaps remain. First, the studies reporting risk-adjusted outcomes have largely been restricted to selected populations, e.g. hospitals, regions or states and for selected years. The most recent study to examine risk-adjusted outcomes at the national level over time was conducted using data between 1950 and 1975 [24]. Second, in the current era of healthcare reform local-level assessments are critical for informing policy for interventions. For example, in regions with low risk-factor prevalence and high risk-adjusted rates, more attention should be paid to improve access to and quality of neonatal care. In those regions with high-risk factor prevalence and low risk-adjusted rates, policy should be focus on interventions to reduce preterm birth and low-birth weight babies. Third, previous studies examining the associations between clinical care and risk-adjusted outcomes are largely based on older datasets, for example Goodman and colleagues use the linked birth-death dataset from 1996 [25]. With the US neonatal mortality rate remaining at persistently high levels, now is an opportune time to examine the contribution of risk factors versus the contribution of clinical care to better understand the lack of progress and continued disparities in newborn survival.

The objective of our study is two-fold. The first is to describe trends and subnational variation in early neonatal mortality, the expected early neonatal mortality based on gestational age (GA) and birth weight (BW), and GA-BW adjusted early neonatal mortality in the US between 1996 and 2006. The second is to quantify the relationship between health system indicators and subnational variation in risk-adjusted mortality.

**Table 1:** Health Service Area (HSA) covariates.

HSA level covariates	
Volume of preterm infants	The number of preterm* infants born by quintile
Neonatal Intensive Care Unit (NICU)	The presence of one or more NICUs in the HSA (binary)
Obstetrician and Gynecologists	The number of ObGyn per 1000 live births in the HSA by quintile
Neonatologists	The number of Neonatologists per 1000 preterm* births in the HSA by quintile
Neonatal Intermediate Care	The availability of Neonatal Intermediate care facilities in the HSA (binary)
NICU beds	The number of NICU beds per 1000 preterm* births in the HSA by quintile among HSAs that contained a NICU

* Preterm was defined as a birth before 34 weeks gestation.

## Methods

### Data and Definitions

We used linked birth and death data from the National Vital Statistics System (NVSS) for the years 1996 to 2006, the latest available year, from the US Centers for Disease Control and Prevention. We used the birth cohort linked data for 1996 to 2003 and created cohort data by linking period-based birth and death data using gestational age, birth weight, year of birth and county of birth for the years 2004 to 2006. We excluded neonates weighing less than 500 g due to differential classification of live-birth status based on weight [26], [27].

**Table 2:** First-stage GA-BW adjustment model for Early Neonatal Deaths among US births (1996–2006).

	Odds Ratio	Coefficient	95% CI	Observations incombined dataset	ENND rate per 1000
**Gestational Age (weeks)**					
<20	26.01	3.258	(3.071 to 3.446)	3,238	538.60
20–21	29.15	3.372	(3.232 to 3.513)	8,845	470.44
22–23	24.93	3.216	(3.101 to 3.331)	38,317	491.74
24–25	6.34	1.847	(1.734 to 1.96)	85,851	181.48
26–27	2.68	0.987	(0.872 to 1.101)	121,716	72.06
28–29	2.05	0.716	(0.603 to 0.83)	195,709	30.20
30–31	1.92	0.652	(0.544 to 0.76)	341,693	16.19
32–33	1.79	0.580	(0.493 to 0.666)	2,469,543	4.88
34–35	1.47	0.383	(0.301 to 0.464)	5,686,001	1.38
36–37	1.02	0.025	(−0.049 to 0.098)	18,296,659	0.52
38–39	*Reference Category*			13,653,407	0.44
≥40	1.33	0.284	(0.169 to 0.399)	3,043,716	0.64
**Birth weight (grams)**					
500–999	162.58	5.091	(4.973 to 5.209)	250,371	202.63
1000–1499	51.65	3.944	(3.832 to 4.057)	321,159	29.82
1500–1999	28.40	3.346	(3.243 to 3.45)	673,060	13.54
2000–2499	10.52	2.353	(2.258 to 2.449)	2,113,385	4.10
2500–2999	3.19	1.159	(1.068 to 1.249)	7,541,329	1.03
3000–3499	1.41	0.347	(0.257 to 0.437)	16,609,112	0.42
3500–3999	*Reference Category*			12,362,927	0.29
4000–4499	1.06	0.059	(−0.093 to 0.212)	3,470,281	0.33
4500–4999	1.96	0.671	(0.412 to 0.931)	542,584	0.61
5000-max	7.81	2.056	(1.678 to 2.434)	60,487	2.07
**Year**					
1996	*Reference Category*			3,848,529	2.39
1997	0.90	−0.103	(−0.173 to −0.033)	3,840,016	2.34
1998	0.94	−0.065	(−0.135 to 0.004)	3,898,581	2.32
1999	0.87	−0.140	(−0.211 to −0.07)	3,914,362	2.28
2000	0.85	−0.160	(−0.232 to −0.089)	4,014,281	2.24
2001	0.82	−0.193	(−0.265 to −0.122)	3,985,449	2.19
2002	0.86	−0.156	(−0.227 to −0.086)	3,979,546	2.21
2003	0.78	−0.251	(−0.323 to −0.179)	4,045,355	2.19
2004	0.77	−0.258	(−0.33 to −0.187)	4,069,108	2.17
2005	0.76	−0.270	(−0.342 to −0.198)	4,109,128	2.12
2006	0.76	−0.277	(−0.349 to −0.205)	4,240,340	2.10
**Constant**		−8.059	(−8.154 to −7.963)		

We purposefully excluded covariates that may be correlated with quality of care, such as socioeconomic status and race. Similarly, the presence of an APGAR score reflects the process of care and thus may be correlated with quality of care. Data on congenital anomalies were also excluded due to inconsistent coding.

**Figure 1:**
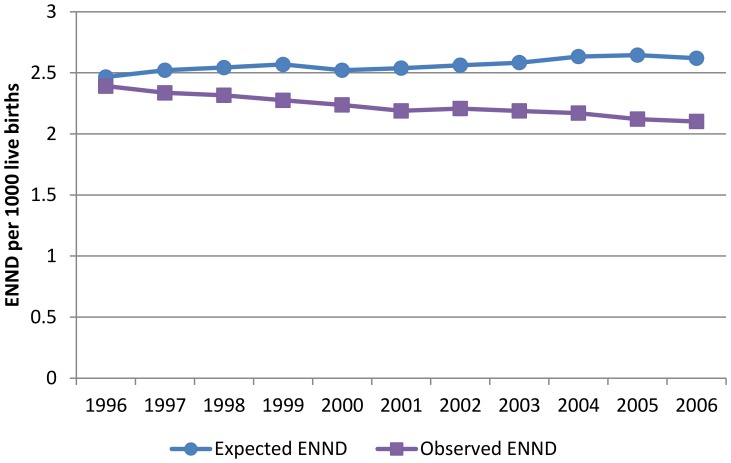
Expected and Observed ENND rate 1996–2006.

The primary outcome of the analysis was early neonatal death (ENND), defined as death occurring within 7 days of birth. We assessed subnational variation by Health Services Area (HSA). HSAs are a single county or cluster of contiguous counties which are relatively self-contained with respect to hospital care [28]. We mapped counties to 802 unique HSAs andcreated indicators for each HSA, including the presence of a NICU, the presence of neonatal intermediate care facilities, and the number of obstetrician-gynecologists and neonatologists (excluding those not primarily involved in clinical practice) using data from the Area Resource File and Physician Masterfile from the American Medical Association (AMA) [29], [30].

**Figure 2:**
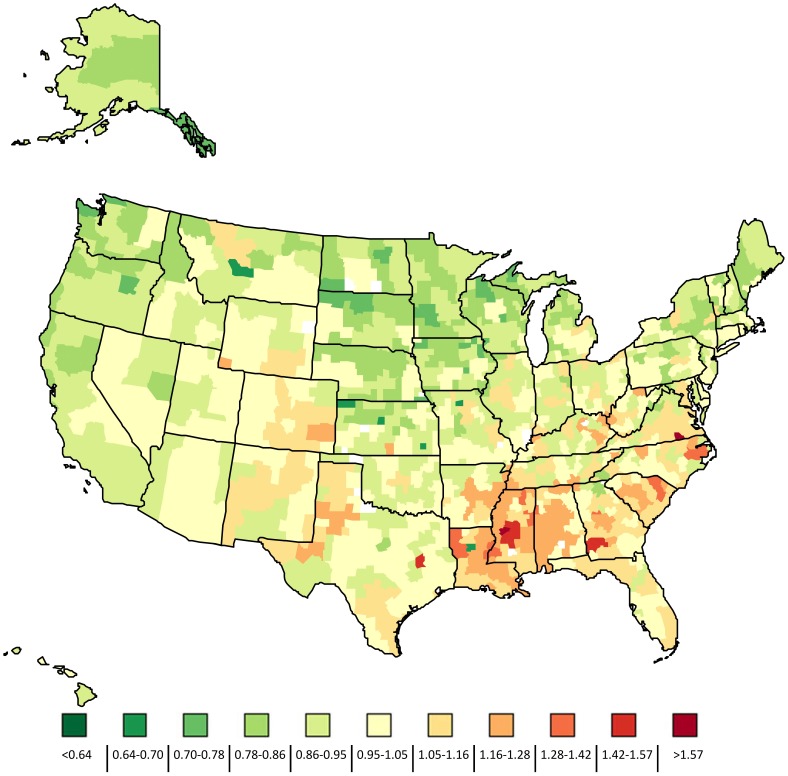
Ratio of the expected early neonatal mortality rate compared to national average by HSA, 2004–2006.

### Statistical Analyses

We developed our first-stage GA-BW adjustment model of ENND estimated using multivariate logistic regression to control for the two primary biological predictors of ENND: gestational age (0–19 weeks, 2 weeks intervals up to 40+ weeks) and birth weight (500 gram intervals from 500 to >5000). We also control for temporal trends using 1996 as the reference year. With the large number of observations (44 million), to facilitate computation, we estimated the model coefficients based on a random sub-sample and then applied the estimated coefficients to the full dataset. We used a random sub-sample of 800,000 observations from each year from 1996 to 2006– a total of 8.8 million records. To ensure that our model wasn’t unduly influenced by regions with a high prevalence of premature births, which may result from the transfer of high risk births, we conducted a sensitivity analysis of the coefficients sampling only from HSAs among the lowest two quintiles of ENND.

**Figure 3:**
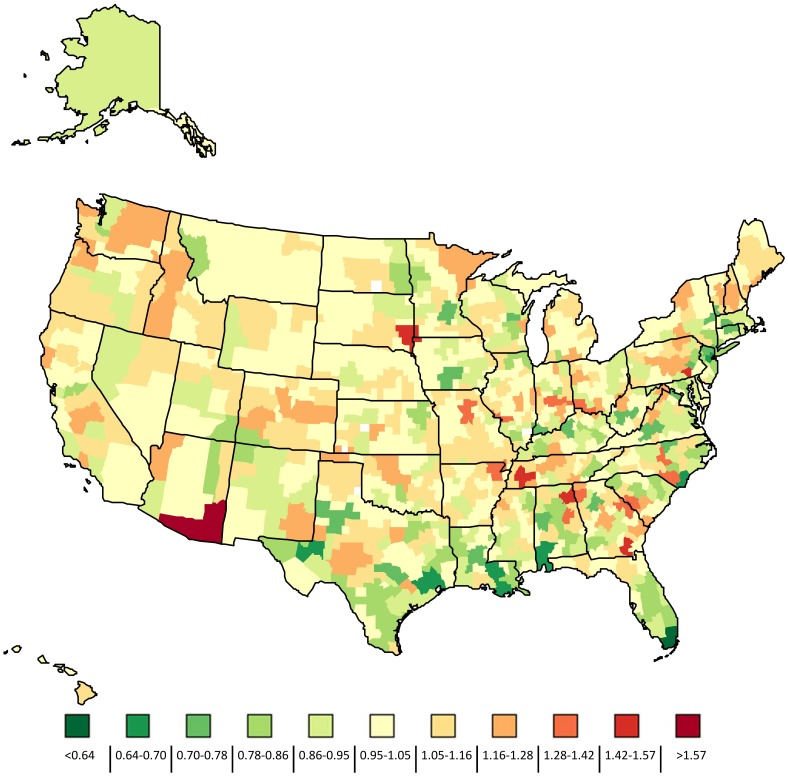
Ratio of the GA-BW adjusted early neonatal mortality rate compared to national average by HSA, 2004–2006.

We used the linear prediction to determine the expected early neonatal mortality rate based on the distribution of gestational age and birth weight for each HSA-year. We regressed the variance of expected early neonatal mortality rate among HSAs on year to assess if there was a relationship between risk factor disparity and time. We also used the prediction for each record as a covariate in a second-stage logistic regression model where we include a random effect by HSA, (assumed to be normally distributed with a mean of zero). The random effect quantifies the remaining variation in early neonatal mortality at the HSA-level after accounting for differences in the distribution of gestational age and birth weight, i.e. it represents the GA-BW adjusted mortality rate. This second stage model was run using three sequential years of pooled data across the time period, i.e. 1996–1998, 1997–1999 to 2004–2006. We regressed the variance of the HSA random intercept on year to assess if there was a relationship between variation in clinical care and time.

**Table 3:** Univarite relationship between HSA-level covariates and GA-BW adjusted early neonatal mortality (2004–2006).

	All births			VLBW		
	OR	95% CI	p	OR	95% CI	p
**NICU**						
No NICU	*Reference*			*Reference*		
NICU	0.791	(0.754 to0.829)	<0.001	0.505	(0.47 to 0.543)	<0.001
**NICU Beds**						
None	*Reference*			*Reference*		
0.51–11.34	0.812	(0.770 to0.857)	<0.001	0.548	(0.507 to0.592)	<0.001
11.34–17.86	0.741	(0.705 to0.779)	<0.001	0.510	(0.473 to0.549)	<0.001
17.86–23.42	0.794	(0.755 to0.834)	<0.001	0.515	(0.478 to0.555)	<0.001
23.42–30.19	0.811	(0.769 to0.856)	<0.001	0.532	(0.492 to0.575)	<0.001
>30.19	0.932	(0.877 to0.991)	0.024	0.597	(0.547 to0.653)	<0.001
**Neonatal intermediate care**						
Not available	*Reference*			*Reference*		
Available	0.883	(0.851 to0.917)	<0.001	0.742	(0.704 to0.782)	<0.001
**ObGyns per 1000 births**						
0–1.27	*Reference*			*Reference*		
1.27–1.86	1.014	(0.928 to1.109)	0.753	0.936	(0.819 to1.069)	0.33
1.86–2.39	0.986	(0.906 to1.073)	0.748	0.830	(0.731 to0.943)	0.004
2.39–3.07	0.912	(0.840 to0.989)	0.027	0.734	(0.649 to0.831)	<0.001
>3.07	0.908	(0.837 to0.984)	0.019	0.751	(0.665 to0.849)	<0.001
**Neonatologists per 1000 preterm births**						
None	*Reference*			*Reference*		
0.75–2.45	0.901	(0.839 to0.966)	0.004	0.764	(0.688 to0.849)	<0.001
2.45–3.47	0.790	(0.718 to0.868)	<0.001	0.601	(0.526 to0.688)	<0.001
3.47–4.82	0.840	(0.776 to0.909)	<0.001	0.598	(0.535 to0.669)	<0.001
4.82–6.56	0.803	(0.748 to0.863)	<0.001	0.505	(0.456 to0.560)	<0.001
>6.56	0.754	(0.719 to0.791)	<0.001	0.479	(0.446 to0.515)	<0.001
**Volume of preterm infants** *						
0–68	*Reference*			*Reference*		
69–186	0.994	(0.814 to1.214)	0.956	0.860	(0.611 to1.210)	0.387
187–480	0.900	(0.746 to1.086)	0.271	0.695	(0.505 to0.957)	0.026
481–1417	0.806	(0.673 to0.966)	0.02	0.462	(0.339 to0.629)	<0.001
>1417	0.715	(0.598 to0.854)	<0.001	0.345	(0.254 to0.467)	<0.001
**Volume and NICU**						
*No NICU*						
0–68	*Reference*			*Reference*		
69–186	0.997	(0.808 to1.231)	0.980	0.897	(0.625 to1.288)	0.557
187–480	0.900	(0.737 to1.099)	0.301	0.773	(0.549 to1.088)	0.140
481–1417	0.894	(0.729 to1.097)	0.282	0.749	(0.530 to1.058)	0.101
>1417	0.793	(0.618 to1.018)	0.069	0.446	(0.304 to0.653)	<0.001
	**All births**			**VLBW**		
	**OR**	**95% CI**	**p**	**OR**	**95% CI**	**p**
*NICU*						
0–68	0.949	(0.521 to1.73)	0.864	1.683	(0.629 to4.503)	0.300
69–186	0.931	(0.669 to1.296)	0.674	0.973	(0.550 to1.720)	0.925
187–480	0.884	(0.709 to1.101)	0.271	0.644	(0.445 to0.933)	0.020
481–1417	0.782	(0.646 to0.947)	0.012	0.452	(0.326 to0.626)	<0.001
>1417	0.710	(0.589 to0.857)	<0.001	0.362	(0.263 to0.500)	<0.001

* Refers to period 2004–2006.

Using the pooled data from 2004 to 2006 (12,439,751 births), we compared the variances of the random effect in the base case model to models where we include along with the linear prediction, fixed effects for a range of HSA-level covariates, such as the presence of a NICU or the volume of pre-term births (Table 1). We separately tested the total volume of births in the HSA to test if volume of preterm births was capturing effects related to HSA size. This allows us to examine what fraction of the residual variation, after accounting for gestational age and birth weight, is explained by these indicators. We repeated this analysis using only very-low birth weight (VLBW) births, defined as less than 1500 grams. Continuous variables were categorized into quintiles with the lowest quintile serving as the reference group.

**Table 4:** Variance in GA-BW adjusted ENN mortality explained by HSA covariates (2004–2006).

	% Variance explained	
	All births	Very Low Birthweight
	>500 g	>500 g & <1500 g
*Univariate*		
NICU	14.3%	45.1%
NICU beds	19.9%	47.4%
Neonatal Intermediate Care	5.5%	16.9%
ObGyn	7.1%	11.2%
Neonatologists	19.8%	53.8%
Volume	17.6%	50.4%
*Multivariate*		
Volume and NICU beds	22.7%	55.5%
NICU and Neonatologists	20.6%	57.8%
Volume and NICU	18.8%	54.8%
Volume, NICU and interaction	19.0%	55.7%
Volume, NICU and Neonatologists	21.0%	58.3%
Volume, Neonatologists and Beds	25.2%	58.7%

## Results

There were 44,015,582 births weighing at least 500 grams from 1996 to 2006. Among these births there were 97,914 ENND which accounted for 71.2% of all neonatal deaths. Table 2 shows the coefficients from the risk-adjustment model based on our random sample of records from 1996 to 2006. We found no significant differences in the coefficients derived from only those HSAs among the lowest two quintiles of ENND. As expected, the highest odds of ENND are among newborns in the lowest gestational age category (<19 weeks) and among those within the lowest birth weight category (500–999 grams).

The odds of ENND, after controlling for birth weight and gestational age, has declined steadily over this time period. There was a statistically significant 15% lower odds of early neonatal mortality in 2000 compared to 1996, after controlling for birth weight and gestational age. By 2005–2006, there was a 24% lower odds of ENND compared to 1996 (Table 2). The observed ENND rate declined from 2.39/1,000 births in 1996 to 2.10/1,000 births in 2006; an overall reduction of 12% (Figure 1). The expected ENND rate provides an estimate of the ENND rate based only on changes in the distribution of gestational age and birth weight, holding the background mortality rate to 1996 levels. In contrast to the observed rate, the expected ENND rate increased steadily to 2.62/1,000 in 2006, reflecting adverse trends in gestational age and birth weight.

We consider spatial patterns to refer to geographic clustering of rates. There were strong spatial patterns in the expected ENND rate (Figure 2). The map of HSA-level variation shows the ratio of the expected ENND rate to the national average for the period 2004–2006. The highest expected ENND rates were found in HSAs located in Mississippi, Georgia, Louisiana, Alabama, and North and South Carolina. Among the highest ENND rates were HSAs comprising Attala, Hinds, Holmes, Leake, Madison, Rankin, Scott and Simpson counties in Mississippi (Ratio = 1.50) and the counties Baker, Calhoun, Clay, Dougherty, Early, Lee, Randolph, Terrell and Worth in Georgia (Ratio = 1.47). The lowest expected ENND rate was in Sioux county, Iowa (Ratio = 0.73). We found a statistically significant positive relationship (R = 0.85, p = 0.03) between the variation in the HSA-level expected ENND rate and calendar year indicating that there are increasing disparities in risk factor prevalence over time.

GA-BW adjusted mortality did not exhibit strong spatial patterns (Figure 3). There was only a slight negative correlation between the expected ENND rate and GA-BW adjusted ENND rate (R = −0.1). The HSAs with the lowest GA-BW adjusted ENND rates were located in Florida, Texas, New Jersey, Louisiana, Alabama and North Carolina. The lowest GA-BW adjusted rates were in the HSAs comprising Broward, Miami-Dade and Monroe counties in Florida (Ratio = 0.63) and Crane, Ector, Reeves, Ward and Winkler counties in Texas (Ratio = 0.64). The GA-BW adjusted rate was highest in a HSA located in Southern Arizona comprising the counties Cochise, Graham, Greenlee, Pima and Santa Cruz (Ratio = 1.68). There was no statistically significant relationship between the variance in GA-BW adjusted early neonatal mortality and calendar year, indicating that there has been no significant change in disparities in GA-BW adjusted mortality over time.

Table 3 presents univariate fixed-effects for selected health system indicators for the 2004 to 2006 period. The presence of a NICU was associated with a 21% lower odds of ENND among all births, and a 51% lower odds among VLBW births. The presence of neonatal intermediate care was also associated with a lower odds of ENND. There was no evidence, however, of a larger reduction in the odds of mortality with increasing quintiles of NICU beds per birth.

Significant reductions in the odds of ENND were only present in HSA’s with at least 2.39 obstetricians-gynecologists per 1,000 births among all births and more than 1.86 obstetricians-gynecologists per 1,000 births among VLBW births. HSA’s with neonatologists present were associated with a significant reduction in the odds of ENND compared to HSAs without neonatologists. There was evidence of a larger effect with increasing quintiles of neonatologists per birth among very low birth weight babies. Higher volumes of preterm births were associated with a reduced risk of death, however these finding were only statistically significant among those HSAs that also contain NICU facilities.

Table 4 presents the percentage of subnational variation in GA-BW adjusted mortality that can be explained by the inclusion of HSA-level fixed effects. In our univariate analyses, the presence of a NICU explained 14.3% and 45.1% of the HSA variation in GA-BW adjusted outcomes among all births and VLBW births respectively. The presence of neonatal intermediate care resources explained 5.5% of the variation in all births and 16.9% among VLBW births. NICU beds, the number of neonatologists and the volume of preterm births explained the highest percentage of the variance; around 20% of the variation in GA-BW adjusted outcomes among all births and around half of the variation among VLBW births. The volume of preterm births was more predictive of outcome than total volume suggesting that HSA size was not driving the relationship between volume of preterm births and risk of ENND.

In our multivariate analysis, preterm volume, the number of neonatologists and the number of NICU beds explained 25.2% of the variance in GA-BW adjusted outcomes among all births and 58.7% of the variance among VLBW births.

## Discussion

Our findings indicate that part of the reason for the lack of progress in reducing neonatal mortality in the United States compared to other countries is that the prevalence of high-risk births has steadily increased. This has offset improvements through clinical care that reduced GA-BW adjusted early neonatal mortality by about one-quarter between 1996 and 2006. As a result, the observed early neonatal mortality rate over the same period declined by only about one-tenth. We found subnational variation in both the prevalence of high risk births and GA-BW adjusted mortality with the variation in high-risk births but not GA-BW adjusted mortality increasing over time. Health system indicators of obstetric and neonatal care explained around one-quarter and more than one-half of the variation in risk adjusted mortality, for all births and VLBW births, respectively.

The reasons for the increasing prevalence and variation in earlier gestational age and lower birth weights are complex. The spatial patterns of high-risk births – with higher prevalence in Southern states such as Mississippi, Georgia, Louisiana, Alabama, North Carolina and South Carolina – mirror other health indicators such as life-expectancy [31] and the prevalence of adult diabetes [32]. This suggests a set of shared determinants that likely include socioeconomic disadvantage, lack of education, poorer nutrition and increased exposure to alcohol, tobacco and illicit drugs, all of which have been shown to be associated with earlier gestational age and lower birth weight [33]. It is also possible poor *in utero* nutrition could be correlated with both poor early neonatal outcomes as well as a higher prevalence of adult conditions such as type II diabetes, as postulated by the fetal origins hypothesis [34]. Other studies indicate that part of the increase in earlier gestational age may be related to early induction of labor and use of cesarean delivery [35], [36].

GA-BW adjusted mortality has improved over time, indicating that the quality of obstetrical and neonatal care is improving. Likely mechanisms of improved obstetric and neonatal care include the expanded use of surfactant replacement therapy [37] as well as improvements in cardiovascular and respiratory treatments of ill newborns [11]. We found a relationship between improved outcomes and the volume of preterm infants which was moderated by the availability of NICU resources. These findings are consistent with a study from California [12] that found that both high volume and high levels of care were associated with lower mortality among VLBW births. Notably, there were 43 HSAs in the highest two quintiles of volume accounting for almost 32,000 preterm births during 2004 to 2006 without NICU facilities. The addition of, or more appropriately reallocation of existing, NICU resources to these HSAs has the potential to reduce ENND.

Consistent with our findings, an earlier national study found that neonatal mortality was not improved with an increased number of neonatologists or NICU beds [25], however, when restricted to VLBW births we found evidence of an increasing effect of more neonatologists on outcomes. There are several possible explanations. Firstly, we used early neonatal mortality rather neonatal mortality as our primary outcome; late neonatal deaths may be less influenced by intensive care resources. Secondly, we considered neonatologists and NICU beds in the context of the volume of preterm births rather than all births. Given only a small proportion of births require ICU resources, the relationship between neonatologists and outcomes may have been confounded in the previous study by using overall volume. Finally, the previous study was based on data from the mid-1990s and the differences with our study may reflect changing relationships over time.

While the increasing prevalence of preterm birth seems to be driving the decline in US rankings, a review of approximately 2000 studies, found only 2 interventions that were effective in preventing preterm birth, namely smoking cessation and progesterone [38]. For indicated preterm deliveries, a previous Cochrane review found that anti-platelet drugs were effective in modestly reducing preterm birth rates and neonatal death among women with risk factors for pre-eclampsia [39]. A meta-analysis of birth spacing and neonatal outcomes showed that interpregnancy intervals shorter than 6 months were associated with 1.4 times the odds of preterm delivery when compared to interpregnancy intervals of 18–23 months [40]; improved education about birth spacing and access to effective contraception may help to reduce early neonatal mortality. As others have noted, even with widespread implementation of these interventions only a small percentage of preterm deliveries would be averted [41]. This highlights the need for further research on effective interventions to reduce the incidence of preterm births and low-birth weight babies.

Effective policy targeted at reducing neonatal mortality will differ depending on the region. Parts of North East Minnesota have a very low expected ENND rate and a GA-BW adjusted rate that is higher than expected. This suggests that policy should target improvements in the quality of care – potentially through the regionalization of birthing facilities. In contrast, in regions in south-eastern Florida, policies targeting smoking cessation, progesterone use for high-risk pregnancies, increases in birth spacing as well as interventions designed to reduce mortality in preterm deliveries are likely to be more effective. Our findings should be interpreted with several limitations in mind. Firstly, our analysis was conducted at a regional not institutional level. This means that while an HSA may contain a NICU, or other resources, we cannot assess whether an individual birth used those resources. Secondly, we were only able to explain a fraction of the variation in GA-BW adjusted mortality. This reflects the fact that we only had relatively crude indicators of quality of care and were not able to capture differences related to factors such as how efficiently resources are being used, training and skills of providers and other aspects of an individual’s process of care. Remaining variation may also be a reflection of differences in the presence of congenital anomalies. The data on congenital anomalies was poorly recorded and inconsistent in the data and we were not able to control for this risk factor. In addition, it’s likely that there are regional differences in the rate of abortion and this will confound our results if pregnancy termination is correlated with unadjusted risk factors for ENND. Finally, as we only had information about risk factors at the time of birth it was not possible to separate out the mortality impacts of obstetrical care versus neonatal care, although we show a stronger relationship between GA-BW adjusted mortality and measures of neonatal care resources compared to obstetric resources.

### Conclusions

A shift towards higher-risk births has offset gains in the quality of obstetric and neonatal care services. Substantial subnational variation in both mortality related to gestational age and birth weight and GA-BW adjusted mortality remain. Efforts to improve neonatal outcomes should consider the relative contributions of risk factors and quality of care at the local level. At the national level, there is some scope to improve early neonatal outcomes through expansion or reallocation of clinical neonatal resources towards regions with high volumes of preterm infants. There is the potential for more significant improvement in outcomes through risk factor prevention; however, the pathways to achieving this are less clear which highlights the need for further research on interventions to prevent preterm birth and low birth weight babies.

## Supporting Information

Table S1 **Random intercept by State, HSA and County.**
(XLSX)
